# Interdependence of Angiogenesis and Arteriogenesis in Development and Disease

**DOI:** 10.3390/ijms23073879

**Published:** 2022-03-31

**Authors:** Ferdinand le Noble, Christian Kupatt

**Affiliations:** 1 Department of Cell and Developmental Biology, Institute of Zoology (ZOO), Karlsruhe Institute of Technology (KIT), Fritz Haber Weg 4, 76131 Karlsruhe, Germany; 2 Institute for Biological and Chemical Systems—Biological Information Processing, Karlsruhe Institute of Technology (KIT), P.O. Box 3640, 76021 Karlsruhe, Germany; 3 Institute of Experimental Cardiology, Heidelberg Germany and German Center for Cardiovascular Research (DZHK), Partner Site Heidelberg/Mannheim, University of Heidelberg, 69117 Heidelberg, Germany; 4 Klinik und Poliklinik für Innere Medizin I, Klinikum Rechts der Isar, Technical University Munich, 81675 Munich, Germany; 5 DZHK (German Center for Cardiovascular Research), Munich Heart Alliance, 80802 Munich, Germany

**Keywords:** angiogenesis, arteriogenesis, blood flow, shear stress, MRTF-A, AAV, endothelial cell shape, sFlt1, Trio

## Abstract

The structure of arterial networks is optimized to allow efficient flow delivery to metabolically active tissues. Optimization of flow delivery is a continuous process involving synchronization of the structure and function of the microcirculation with the upstream arterial network. Risk factors for ischemic cardiovascular diseases, such as diabetes mellitus and hyperlipidemia, adversely affect endothelial function, induce capillary regression, and disrupt the micro- to macrocirculation cross-talk. We provide evidence showing that this loss of synchronization reduces arterial collateral network recruitment upon arterial stenosis, and the long-term clinical outcome of current revascularization strategies in these patient cohorts. We describe mechanisms and signals contributing to synchronized growth of micro- and macrocirculation in development and upon ischemic challenges in the adult organism and identify potential therapeutic targets. We conclude that a long-term successful revascularization strategy should aim at both removing obstructions in the proximal part of the arterial tree and restoring “bottom-up” vascular communication.

## 1. Introduction

Arterial networks are tree-like hierarchically branched structures that distribute blood flow via resistance-sized arteries and arterioles of gradually decreasing diameter to the distal capillary network, which is the exchange area for oxygen and nutrients. The arterial branching architecture and lumen dimensions are optimized to allow for efficient blood flow distribution while minimizing transport costs [1]. Continuous information transfer along the vascular tree is crucial for design optimization upon changes in hemodynamic conditions and organ metabolism. The formation of arterial collateral networks as occurs in ischemic cardiovascular diseases represents a specialized design optimization solution aimed at restoring flow delivery to compromised regions [2]. How collaterals are formed and how to selectively target their growth is an outstanding question in the field. Recent studies have shown that efficient induction and maintenance of stable arterial collateral networks requires precise retrograde information transfer between the microcirculation and the upstream arterial network in line with design optimization principles [3]. Unfortunately, patients with ischemic CVD typically have underlying risk factors that interfere with the retrograde information transfer process. Given the interdependence of arteries and capillaries for vessel network design optimization, we propose that successful collateralization strategies should consider microcirculation functionality. We highlight some new insights and collateralization approaches based on recent advances in understanding arterial network growth.

Conspicuously, analysis of arterial growth and adaptation to exercise, and arterial development during embryogenesis and neonatal stages, revealed in striking similarity, that arteries obtain their structure through inward growth. This is according to a “bottom-up” principle involving substantial retrograde communication against the direction of blood flow [3,4,5]. Taking the present clinical focus on removing obstructions in the upstream part of the arterial tree into account—through the insertion of a stent or bypass surgery—it becomes clear that the bottom-up principle is not heeded to a vast extent. In particular, most ischemic CVD patients harbor underlying risk factors, such as diabetes, hypertension, hyperlipidemia, or genetic diseases that adversely affect endothelial function and microvascular structure, prohibiting retrograde communication and structural optimization. Indeed, clinical evidence suggests that long-term end-organ function and survival upon ischemic insults not only requires removing the upstream arterial blockage with a stent, but also careful nurturing of the peripheral microcirculation [6]. We will advocate retrograde “bottom-up” communication to stimulate arterial growth, both of arterial bypass collaterals and of microvascular arterioles, as adjuvant therapy to the conventional revascularization strategies.

## 2. An Underestimated Problem—Capillary Rarefaction in Vascular Disease

The relevance of the microcirculatory vessel compartment may not seem obvious for ischemic heart disease, one of the most prevalent factors reducing health in Western societies and beyond. Becoming apparent as acute myocardial infarction due to atherosclerotic plaque rupture or hibernating myocardium due to prolonged plaque progression, coronary artery disease is treated as large artery disease by interventional or surgical means. In that perspective, the relevance of the interdependence of arteries and capillaries is easily underestimated. However, cardiovascular and genetic risk factors, which are responsible for the majority of cardiac events, are also affecting microvascular integrity. For example, capillary rarefaction in organs such as the eye and the kidney precede and predict macro vessel obstruction [7]. Capillary rarefaction in the coronary circulation is a hallmark of diabetes mellitus in small [8] and large animal models [9]. For this cardiovascular risk factor, revascularization therapy appears notoriously hard to apply via cardiac intervention studies [10], not least since microvascular hypodensity caused by capillary and arteriolar rarefaction, precludes a flow increase upon revascularization therapy. Hyperlipidemia, in particular hypercholesterinemia, is a major causative factor in atherogenesis and activation of inflammation [11]. Inflammatory processes induce rarefaction of small vessels, i.e., arterioles and capillaries [12], which profoundly alter the coronary flow pattern. Lipid-lowering pharmacological interventions, then resolve not only endothelial dysfunction, but also capillary rarefaction inflicted by chronic ischemia in a rabbit hindlimb model [13]. Hypertension, another major cardiovascular risk factor, has been associated with capillary and arteriolar rarefaction [14]. Sudden experimental onset of hypertension by transverse aortic banding (TAC) may induce a massive macrophage-driven hypertrophy, concomitant with induction of fibrosis and capillary rarefaction, which are, at least in part driven by miR21 [15,16]. Puzzling enough, hypertension is a common side effect of Vascular Endothelial Growth Factor (VEGF) neutralizing biologics, exemplifying the relevance of the microcirculation for hemodynamic homeostasis [17].

## 3. Angiogenic Vessel Building—Building back Better-Perfused Capillaries and Arterioles

**Figure 1:**
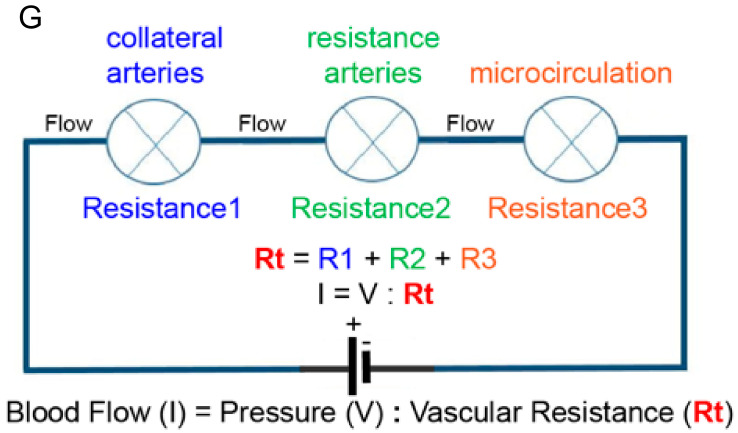
Schematic illustration of an arterial network indicated as electrical resistances coupled in series to a battery. In electrical terms: I (current) = V (voltage) divided by R (resistance), in biological terms Amount of Blood Flow = Pressure difference divided by Peripheral Resistance. The collateral arteries are upstream and coupled in series with the microcirculation. Therefore, changes in the resistance of the microcirculation affect the blood flow through the network. Lowering resistance R3 (microcirculation) will result in lower total resistance (Rt). As a consequence, at a given pressure difference (P), more flow will go through the arterial network. Subsequently, the upstream arterial collaterals (Resistance 1) will become exposed to more flow and shear stress levels, and increased shear stress stimulates outward lumen remodeling of collateral arteries. Diabetes and hypertension interfere with this feedback system.

If capillaries and arteries fall victim to cardiovascular risk factors, which options are biologically available to counteract this tendency? A look into vessel physiology is warranted. Oxygen delivery requires lumenized and perfused vessel segments, not blind-ending nonperfused angiogenic sprouts. It is therapeutically relevant to generate arterioles with a patent lumen that can carry blood flow. Titrating lumen diameter is a very important aspect. According to Poiseuille’s law, blood flow through a vessel is proportional to diameter to the power four [18]. Hence a two-fold increase in diameter predicts a 16-fold increase in flow transporting capacity at a given pressure (Figure 1).

Focusing on the bottom of the “bottom-up” principle, lumen formation ex nihilo is not a trivial process, and intense investigation has proposed several models, such as fusion of intracellular vacuoles [19], cord hollowing, and inverse blebbing [20]. Although these models nicely explain how a nonperfused angiogenic sprout generates its initial lumen, little is known how endothelial cells behave and rearrange to obtain a larger lumen during capillary maturation, later arteriologenesis, or how such diameter adaptation process can be stimulated in the context of a therapeutic strategy.

Conventional wisdom leaves a void here and suggests that the transition of a growing arteriole with a small diameter into a large caliber arteriole with a sizeable diameter occurs upon the blood flow driven change in a number of endothelial cells lining the arterial lumen [21,22]. An increase in flow promotes endothelial cell proliferation and migration collectively facilitating the transition of a small caliber vessel segment, with only a few endothelial cells, into a larger caliber arterial segment, with many endothelial cells. Flow activates the Akt-PI3-kinase signaling a pathway responsible for endothelial proliferation. In addition, flow activates the BMP-ALK-Smad signaling pathway, which restricts flow-induced Akt activation and promotes endothelial quiescence [21,23,24,25,26,27].

Attempting to fill the void between formation of a capillary sprout and its growth and integration into a perfused microvascular network, we recently described an alternative, flow-independent model, involving the enlargement of arteriolar endothelial cells, which resulted in the formation of large diameter arterioles [4]. Endothelial enlargement requires the GEF1 domain of the guanine nucleotide exchange factor Trio and activation of Rho-GTPases Rac1 and RhoG in the cell-cell junction region of endothelial cells. Cell domain specific activation of F-actin cytoskeleton remodeling events, and myosin-based tension at junction regions, provide physical forces for a structural enlargement of individual endothelial cells. Activation of Trio in developing arteries in vivo involves precise titration of the Vegf signaling strength in the arterial wall. Interestingly this signaling strength can be titrated by soluble Vegf receptor-1 (sFlt1). Moreover, this study suggests that sFlt1 may be used as a vehicle to deliver a physiologically relevant dosage of Vegf sufficient to stimulate endothelial cell enlargement while avoiding vascular leakage and unproductive angiogenesis, which are typically seen in Vegfa transgenic approaches [4].

The role of vascular sFlt1 acting as a rheostat to control Vegf signaling strength in the context of arterial endothelial cell enlargement may explain some of the pro-arteriogenic effects observed with the Flt1 specific ligands Vegfb and Plgf [28,29,30,31]. Vegfb acts as a cardiac-specific stimulator of arteriogenesis, yet the precise reason for this organ specificity is unclear. One explanation is based on the distribution of Flt1 and Kdr in and around the coronary vasculature. Assuming that Vegfb drives arteriogenesis by competing Vegfa away from Flt1, subsequently triggering Vegfa-Kdr mediated signaling, the tissue distribution of both Kdr and Flt1 may explain where Vegfb initiates the arteriogenesis response. In the coronaries, Flt1 and Kdr only colocalize in the most distal areas and capillaries, whereas coronary arteries in the proximal part express only Flt1. Based on the juxta positioning of Flt1 and Kdr, it is therefore conceivable that arterial enlargement and diameter increases commence in precapillary arterioles [29]. An increase in diameter lowers vascular resistance thereby attracting flow toward the distal regions. This simultaneously augments shear stress to promote outward lumen remodeling in the feed arteries, thereby augmenting blood flow conductance of the entire arterial network [18,29].

Deriving instructions from the observations in development and disease modeling in small and large animals, therapeutic angiogenesis has been applied before using protein and DNA wrapped in non-viral or viral vectors. Despite high pro-angiogenic potency and multiple clinical trials, VEGF-A has not been found to clearly improve ischemic heart function [32]. Owing to its powerful sprouting potential, VEGF-A temporarily increases capillarization up to hemangioma formation [33], without, however, providing mature microvascular networks. This signals back to conductance vessels sufficiently to induce (lumen diameter) growth of the conductance vessels [34]. For these reasons, VEGF-B may be used to greater therapeutic avail, thereby fine-tuning VEGF-A bioavailability by competition at Flt1, the weaker pro-angiogenic receptor, in effect releasing VEGF-A.

## 4. Capillary Maturation and De Novo Arteriologenesis—Linking Angiogenesis and Arteriogenesis

As outlined above, angiogenesis is essential to counteract capillary rarefaction, induced by three of five factors contributing most significantly to cardiovascular disease manifestations. However, evidence in preclinical and clinical studies revealed that stimulation of this process itself does not suffice to improve flow into an ischemic muscle area (reference), particularly in diabetic large animals [9]. Thus, an orchestrated sequence of sprouting of capillaries, potentially supported by the destruction of the surrounding extracellular matrix via Angiopoietin 2, is to be followed by a second phase, namely the recruitment of mural cells such as pericytes and smooth muscle cells for the function connection of capillaries to collaterals. Indeed, a couple of loss-of-function phenotypes indicate the existence of such a bottom-up vessel growth:

Lack of PDGF-B results in a hypercirculatory dilative heart and conductance vessel phenotype contrasted by capillary rarefaction [35], similar to overexpression of Angiopoietin-2, which counteracts pericyte-recruitment and vessel maturation driven by Angiopoietin-1 [36]. These findings indicate that in addition to initial capillary growth, subsequent stabilization by pericyte attachment is required for lasting microvessel structures which may be nurtured by growing collaterals. Thus, stabilization of growing capillaries and mural cell attachment in principle induce arteriogenesis. Of note, microvessel growth-dependent arteriogenesis is lost when the pericytes are intentionally detached, e.g., by Ang-2 overexpression [3]. Accordingly, Notch3 receptor mutations leading to hypomorphic activity and mural cell detachment (CADASIL disease) [37] also appear to provide shrinking of arteriolar and capillary beds leading to brain damage. In a zebrafish model, we have shown that Dll4 activating Notch stabilizes the branching pattern and prohibits aberrant angiogenic sprouting in the developing zebrafish [38]. It should be noted that prohibition of aberrant sprouting is also achieved independently from mural cells, e.g., fostering VE-cadherin-dependent endothelial cell contacts via sphingosin-1-phosphate interacting with its receptor S1pr1 [39].

Conversely, combining VEGF-A with maturation factors such as PDGF-B [40], a pericyte attractant factor, or angiopoietin-1, a microvessel stabilizing factor generated by pericytes themselves [41], suffices to improve flow into the ischemic muscle. Although formation of stabilized and mature capillary beds is likely to be followed by collateral growth, this interdependence is not trivial. Of note, arteriogenesis, forming a conductance vessel network out of preexisting collaterals, is distinct of de novo arteriologenesis, or formation of new arterioles in the microcirculation. Importantly, both processes may occur simultaneously. The molecular and cellular bases of the latter process are poorly understood but evidence is emerging showing similarities to the formation of arteries during early embryogenesis. Recruitment of native collaterals has been well documented and involves shear stress-driven activation of inflammatory processes and activation of monocyte and macrophage migration into the arterial wall collectively promoting outward remodeling, diameter growth, of the native collaterals. However, not all organs possess native collaterals. For example, the mouse heart is not equipped with preexisting collaterals at adulthood, and in such instances, de novo arteriologenesis is the predominant mode responsible for collateralization [42].

## 5. Arteriogenesis in the Developing Vasculature

Recent observations from developing arterial networks during embryogenesis and neonatal stages indicate that arterial trees form in reverse order. Initial arterial endothelial cell differentiation occurs outside of arterial vessels, and these pre-artery endothelial cells then build trees by following a migratory path from smaller into larger arteries, a process guided by the forces imparted by blood flow [5]. Endothelial cells polarize and subsequently migrate against the direction of blood flow, and thereby contribute to the growth and enlargement of the upstream arterial vessel segments. During artery formation, VEGF and Notch signaling act in a common pathway to induce arterial identity in endothelial cells. Notch and DLL ligands are furthermore important for differentiation, physiology, and function of vascular smooth cells in the arterial wall. Arterial endothelial cells use mechanoreceptors to sense the direction of blood flow followed by polarization and migration against this direction involving among others, the APJ, the Eng/Alk1/SMAD4, and DACH1/CXCL12/CXCR4 signaling pathways [5]. These observations from developing arterial networks clearly provide a cellular and molecular substrate for retrograde communication. Yet, it is unclear as to what extent these processes are active or can be re-activated in mature arterial networks in the adult with arterial walls consisting of several layers of smooth muscle. In mice application of CXCL12 can stimulate reassembly of arteries upon myocardial infarction. However, clinical trials thus far failed to demonstrate clinical efficacy [42].

Several studies have shown that in the brain and hindlimb, pre-existing arterial collateral networks develop prior to birth during critical phases of embryonic development [43]. In the brain pre-existing collateral number is highest at the time of birth after which it slowly decreases with age. Hypertension and diabetes accelerate the regression of pre-existing collaterals. In contrast, exercise, most likely via activation of shear stress-dependent mechanisms, prevents or reduces the regression of pre-existing collaterals [44]. Analysis of several inbred mouse strains has shown that arteriogenic capacity, in particular the extent of the pre-existing arterial collateral networks, differs greatly between the analyzed backgrounds suggesting a genetic component in the regulation of this process [38]. Mouse strains with small infarct size upon MCA occlusion showed significantly up to four-fold more and larger pre-existing collaterals when compared with mouse strains showing a relatively large infarct upon MCA occlusion [45]. Genetic linkage studies subsequently correlated the collateral network variation with Rabep2, a regulator of vesicular trafficking wherein cell surface receptors are internalized, and VEGFR2 signaling [46]. The VEGFA-VEGFR2/KDR signaling contributes importantly to the formation of pre-existing collaterals [43,47]. During embryogenesis, reduction of either the ligand or the receptor during the narrow time-window of collaterogenesis reduces collateral formation thus collateral number and diameter in the adult.

At present several known angiogenic proteins significantly impact the formation of collaterals and the extent of pre-existing collateral networks in pia and skeletal muscle including VEGFA, VEGFR2, Dll4, Notch, ADAM10, ADAM17, Gja4, Gja5, and molecules implied in macrophage behavior including Egln1 and NFkB1 [36,40,41,42]. Gja5, also known as connexin 40, is a gap junction protein that mediates electrical communication between endothelial cells thereby allowing vasodilatory signals that are initiated in the distal part of the microcirculation to travel to the proximal–feeding arterioles [48]. In this way, flow can be efficiently routed to the metabolically active regions. Dll4 and Notch are best known for their role in regulating sprouting angiogenesis downstream of VEGF signaling. Genetically or pharmacologically interfering with Dll4-Notch promoted formation of pre-existing collateral in part by enhancing arteriolar branching during late embryonic development [49]. However, in arterial occlusion models, the ischemic outcome did not improve. This was attributed to a defect in capillary functionality, vessel leakage, and impaired flow-induced outward remodeling of arterioles [49]. Perhaps a more physiological—non invasive—way to promote VEGF-Kdrl signaling is to reduce the ambient oxygen tension by moving from sea level to high altitude. As nicely demonstrated by the group of Jim Faber, high altitude rodents such as guinea pigs and deer mice featured a much higher number of collaterals than lowlander species, and were much better protected against cerebral ischemia [50]. The question then is how can lowlanders benefit from this vascular growth potential?

## 6. How Will Distal Microvessel Growth Induce Proximal Collateral Growth?

**Figure 2:**
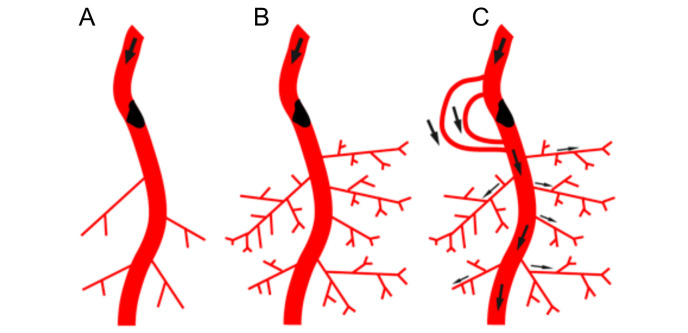

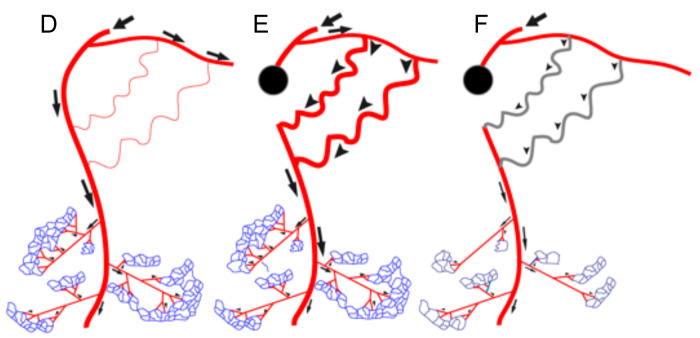
Interdependence of arteriogenesis and angiogenesis in healthy and arteriosclerotic vessels. (**A**): Schematic representation of the arterial network. Arterial occlusion disrupts blood flow to the microcirculation. (**B**): In the presence of the proximal occlusion, stimulation of angiogenesis in the microcirculation will not result in restoring peripheral blood flow. (**C**): Restoring blood flow upon occlusion requires the recruitment of native collaterals or de novo formation of collaterals that can bypass the occlusion in the arterial network. (**D**): Schematic representation of the arterial network in the leg with native arterial collaterals. Flow distribution indicated by arrows. (**E**): Occlusion in the main feed artery results in rerouting of blood flow and flow driven outward remodeling of pre-existing native arterial collaterals (arrowheads). (**F**): Diabetes and hypertension associated with rarefaction of small arterioles and capillary networks (indicated in dark blue) and impaired (flow driven) outward lumen remodeling (arteriogenesis) of arterial collateral networks (impaired collaterals in grey). In this scenario, only targeting upstream collaterals is not sufficient to achieve increased oxygen and nutrient delivery to the periphery. The perfusion of the microcirculation needs to be improved as well.

Since the seminal work of Wolfgang Schaper, direct growth of conductance vessels seemed to be an overarching goal: build the large roads, and the small networks will follow. Arteriovenous shunts providing an unprecedented level of shear stress locally to the vessel wall proved to be the optimal stimulus, the growth potential of which none of the classical or un-canonical growth factors could match [51]. Local inflammation, driven by MCP-1 responsive macrophages [52,53] induces arterial growth, which benefitted most from Il10-mediated M2-subtype polarization, but not from dexamethasone-inhibition of macrophage activation [54,55]. This is a relevant distinction since unselective macrophage-activation (e.g., by MCP-1) might accelerate atherosclerosis rather than solving its consequences, such as macrovascular obstruction [53]. However, even in this conductance-vessel centered model, microvessel networks may play a decisive role in providing pressure gradients required for collateral growth, the absence of which being prohibitive for this process (Figure 2).

## 7. Angiogenesis, Maturation, and Arteriogenesis in Therapeutic Approaches

Are ischemia- and HIF1α-driven factors without alternative, when balanced angiogenesis/arteriogenesis is at stake? Recent observations by us and others have revealed that myogenic factors such as myocardin-related transcription factors (MRTFs), which in myocytes act as cofactors of SRF to keep up specific muscle protein stoichiometry, e.g., upon mechanosensing via the Rho-A pathway [56,57,58]. Of note, therapeutic agents derived from this pathway such as forced expression of MRTF-A or its depressor Thymosin ß4 (scavenging globular actin, which keeps MRTF-A in the cytosol), also provide strong vasoactive signals, synchronizing muscle with vessel growth or maintenance. Interestingly, the secreted factors found after MRTF-A activation are capable of providing capillary stabilization in addition to growth, indicating a program that may secure blood supply during increased demand [3]. Chronic ischemia reduces muscle regeneration and thereby RhoA-signaling dependent vessel maintenance. Indeed both MRTF-A and Thymosin ß4, when applied via a myotropic AAV9 vector, provide capillary growth and maturation in the peripheral and coronary microcirculation. Moreover, unless disrupted by Ang2, MRTF-A provides conductance vessel growth, highlighting the relevance of microvessel maturation for arteriogenesis [3].

**Figure 3:**
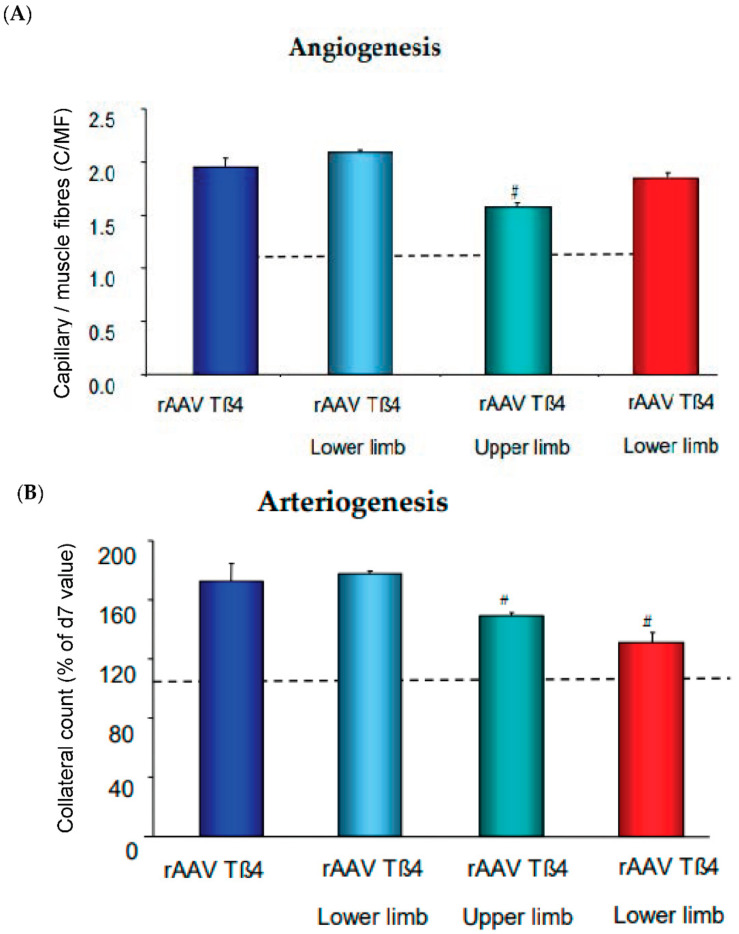
Angiogenesis and arteriogenesis upon recombinant AAV (rAAV) encoding for Thymosin ß4 (Tß4). (**A**) Capillary/muscle fiber ratio (C/MF) after application of rAAVTß4 either whole limb (retrograde venous infusion) or lower limb (intramuscular injection) with or without L-NAME coapplication. (**B**) Collateral count (% of d7 level) at d28. Dotted lines are control group levels. For methods cf. [22].

Of note, the AAV-Tß4 approach in rabbits subjected to femoral artery excision improved arteriogenesis even when only locally applied to the ischemic lower limb. However, this was significantly less effective in increasing flow, when applied directly to the upper limb, i.e., the collateral growth area. Microvascular growth is signaling backward to collaterals to provide more flow, absent macrovessel obstruction. Since shear stress increase would be a likely candidate transmitting this growth signal, we applied L-NAME to inhibit its main effector, nitric oxide formation (Figure 3). Co-application of the NO-inhibitor abrogated solely arteriogenesis, but not angiogenesis. This finding indicates that flow-mediated vasodilation is a powerful signal for arteriogenesis, provided the endothelium is capable of forming the autacoid and is not impaired by atherosclerotic risk factors. To date, it is unclear whether other signals specifically enhancing arterial growth by targeting arterial endothelium, e.g., Notch [59], can therapeutically be utilized for arteriogenesis in addition to microvessel growth. Another potentially promising strategy consists of interference with microRNA regulation which may aid in overcoming negative feedback mechanisms in chronic ischemia of muscles and heart [60,61,62].

## 8. The Concept of Retrograde Communication and Arterial Growth

**Figure 4:**
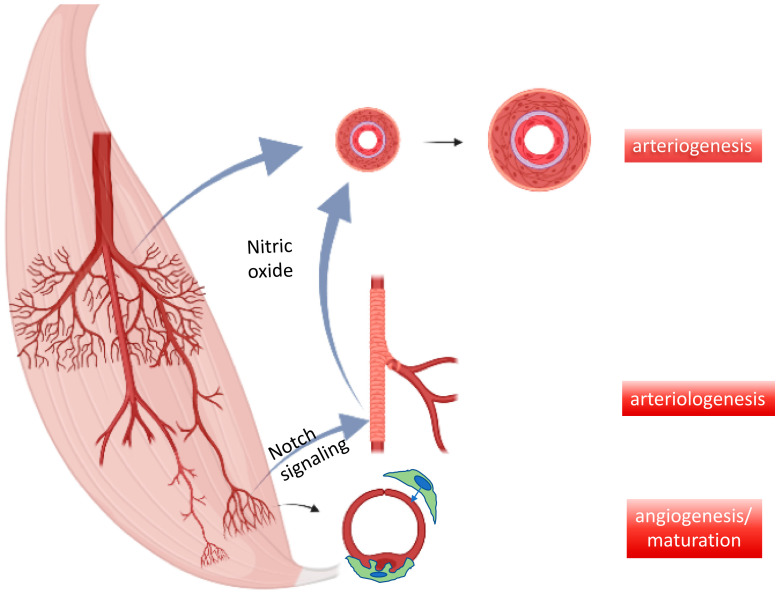
Interdependence of micro- and macrovessel growth. Key mechanisms of vessel growth in developing and adult organisms include capillary formation and stabilization by pericytes (=maturation) as well as resistance control by newly added smooth muscle cells to arteriolar endothelial cells (arteriologenesis), e.g., via Notch signaling. These functional units feedback demand to existing conductance vessels, e.g., via nitric oxide-mediated vessel dilation and subsequent growth (=arteriogenesis).

Retrograde communication has long been recognized in the context of exercise-induced vascular adaptation. Arterial networks dynamically adapt arterial branching patterning, vessel number, and lumen dimensions to exercise-induced changes in organ metabolism. These functional and structural alterations in the arterial network allow precise fine-tuning of flow delivery with the new level of metabolic activity of the organ it innervates. Metabolically active tissue regions are typically close to or in direct contact with the distal parts of the arteriolar microcirculation and the capillary network. In the exercise scenario, the stimulus for arterial network remodeling originates from the change in metabolic status of active cells in tissues surrounding the distal part of the microcirculation. A local mismatch between flow delivery and the new metabolic demand triggers the release of vascular growth factors and vasodilators which promote angiogenesis and expansion of the capillary network. They furthermore reduce vascular resistance in the periphery of the network to attract flow (cf. Figure 4). The local change in resistance and subsequent changes in hemodynamic factors ignite adaptive vascular remodeling responses that are propagated toward the more proximal parts of the arterial network. In these more proximal domains, increases in arterial lumen diameter and optimization of branching architecture augment blood flow conductance thereby increasing oxygen and nutrient delivery to the areas with the increased metabolic demand. The extent of the arterial lumen remodeling is a function of the amount of blood flow, the local shear stress setpoint, and the duration of the stimulus. Whereas an acute increase in flow causes functional endothelium-dependent vasodilation, long term upregulation in flow promotes structural outward arterial lumen remodeling, a process involving rearrangement of endothelial cells and vascular smooth muscle cells around an anatomically-structurally larger lumen. Conversely, restricting exercise and decreasing tissue metabolism and oxygen requirement results in pruning or rarefaction of microvessels, inward arterial lumen remodeling, and an overall reduction of blood flow conductance.

The lessons learned from exercise scenarios is that the initial trigger provoking the arterial network remodeling is caused by a change in resistance to flow at the pre-capillary to capillary level. Metabolic alterations induce the release of specific vascular growth factors (VEGF, angiopoietins), vasoactive metabolites (lactate, adenosine, Vegf), vasodilators (a.o. nitric oxide), as well as retrograde transfer—from the distal to proximal—of vasodilatory responses through electrical coupling in the vessel wall (connexins). Lower resistance in the distal areas will not only attract flow to this region but will also augment flow velocity and shear rates in the more proximal parts that feed the distal region. Increases in shear rates are by themselves a stimulus for dilation or—pending the duration of the stimulus—outward arterial remodeling. Hence the changes in the distal part are communicated to the proximal segments via changes in flow and translated into a gain of lumen dimension. Several risk factors for adverse vascular remodeling such as diabetes alter metabolism and expression of vascular growth factors. Thus they are most likely to affect retrograde communication efficiency.

In summary, microvessel growth requires maturation by the attraction of mural cells to provide long-lasting, functional capillary beds. In addition to providing blood to previously ischemic tissue, microvessel networks are capable of backward signaling to enlarge collaterals via nitric oxide. This interdependence can be exploited therapeutically at various entry points. Whether currently investigated vectors, such as AAVs or AdVs, and delivery systems, such as intramuscular or locoregional applications, hold up to newer developments such as modified RNA-carrying lipid nanoparticles, remains to be seen. However, it appears safe to state that neglecting microvessel network formation and maturation may lead to incomplete and unbalanced vessel growth, which is unlikely to improve the function of ischemic muscle tissue.

## Data Availability

Not applicable.

## References

[1] Sherman T.F. (1981). On connecting large vessels to small. The meaning of Murray’s law. J. Gen. Physiol..

[2] Faber J.E., Chilian W.M., Deindl E., van Royen N., Simons M. (2014). A brief etymology of the collateral circulation. Arter. Thromb. Vasc. Biol..

[3] Hinkel R., Trenkwalder T., Petersen B., Husada W., Gesenhues F., Lee S., Hannappel E., Bock-Marquette I., Theisen D., Leitner L. (2014). MRTF-A controls vessel growth and maturation by increasing the expression of CCN1 and CCN2. Nat. Commun..

[4] Klems A., van Rijssel J., Ramms A.S., Wild R., Hammer J., Merkel M., Derenbach L., Préau L., Hinkel R., Suarez-Martinez I. (2020). The GEF Trio controls endothelial cell size and arterial remodeling downstream of Vegf signaling in both zebrafish and cell models. Nat. Commun..

[5] Red-Horse K., Siekmann A.F. (2019). Veins and Arteries Build Hierarchical Branching Patterns Differently: Bottom-Up versus Top-Down. Bioessays.

[6] Yin H., Arpino J.-M., Lee J.J., Pickering J.G. (2021). Regenerated Microvascular Networks in Ischemic Skeletal Muscle. Front. Physiol..

[7] Rosenson R.S., Fioretto P., Dodson P.M. (2011). Does microvascular disease predict macrovascular events in type 2 diabetes?. Atherosclerosis.

[8] Olivotto I., Oreziak A., Barriales-Villa R., Abraham T.P., Masri A., Garcia-Pavia P., Saberi S., Lakdawala N.K., Wheeler M.T., Owens A. (2020). Mavacamten for treatment of symptomatic obstructive hypertrophic cardiomyopathy (EXPLORER-HCM): A randomised, double-blind, placebo-controlled, phase 3 trial. Lancet.

[9] Hinkel R., Hoewe A., Renner S., Ng J., Lee S., Klett K., Kaczmarek V., Moretti A., Laugwitz K.L., Skroblin P. (2017). Diabetes Mellitus-Induced Microvascular Destabilization in the Myocardium. J. Am. Coll. Cardiol..

[10] Schoos M.M., Dangas G.D., Mehran R., Kirtane A.J., Yu J., Litherland C., Clemmensen P., Stuckey T.D., Witzenbichler B., Weisz G. (2016). Impact of Hemoglobin A1c Levels on Residual Platelet Reactivity and Outcomes After Insertion of Coronary Drug-Eluting Stents (from the ADAPT-DES Study). Am. J. Cardiol..

[11] Silvestre-Roig C., Braster Q., Wichapong K., Lee E.Y., Teulon J.M., Berrebeh N., Winter J., Adrover J.M., Santos G.S., Froese A. (2019). Externalized histone H4 orchestrates chronic inflammation by inducing lytic cell death. Nature.

[12] Ziegler T., Bähr A., Howe A., Klett K., Husada W., Weber C., Laugwitz K.-L., Kupatt C., Hinkel R. (2018). Tβ4 Increases Neovascularization and Cardiac Function in Chronic Myocardial Ischemia of Normo- and Hypercholesterolemic Pigs. Mol. Ther..

[13] Kureishi Y., Luo Z., Shiojima I., Bialik A., Fulton D., Lefer D.J., Sessa W.C., Walsh K. (2000). The HMG-CoA reductase inhibitor simvastatin activates the protein kinase Akt and promotes angiogenesis in normocholesterolemic animals. Nat. Med..

[14] Camici P.G., Tschöpe C., Di Carli M.F., Rimoldi O., Van Linthout S. (2020). Coronary microvascular dysfunction in hypertrophy and heart failure. Cardiovasc. Res..

[15] Ramanujam D., Schon A.P., Beck C., Vaccarello P., Felician G., Dueck A., Esfandyari D., Meister G., Meitinger T., Schulz C. (2021). MicroRNA-21-Dependent Macrophage-to-Fibroblast Signaling Determines the Cardiac Response to Pressure Overload. Circulation.

[16] Hinkel R., Ramanujam D., Kaczmarek V., Howe A., Klett K., Beck C., Dueck A., Thum T., Laugwitz K.L., Maegdefessel L. (2020). AntimiR-21 Prevents Myocardial Dysfunction in a Pig Model of Ischemia/Reperfusion Injury. J. Am. Coll. Cardiol..

[17] Yin G., Zhao L. (2021). Risk of hypertension with anti-VEGF monoclonal antibodies in cancer patients: A systematic review and meta-analysis of 105 phase II/III randomized controlled trials. J. Chemother..

[18] Zhao E., Barber J., Sen C.K., Arciero J. (2022). Modeling acute and chronic vascular responses to a major arterial occlusion. Microcirculation.

[19] Kamei M., Saunders W.B., Bayless K.J., Dye L., Davis G.E., Weinstein B.M. (2006). Endothelial tubes assemble from intracellular vacuoles in vivo. Nature.

[20] Gebala V., Collins R., Geudens I., Phng L.K., Gerhardt H. (2016). Blood flow drives lumen formation by inverse membrane blebbing during angiogenesis in vivo. Nat. Cell Biol..

[21] Jin Y., Muhl L., Burmakin M., Wang Y., Duchez A.C., Betsholtz C., Arthur H.M., Jakobsson L. (2017). Endoglin prevents vascular malformation by regulating flow-induced cell migration and specification through VEGFR2 signalling. Nat. Cell Biol..

[22] Franco C.A., Jones M.L., Bernabeu M.O., Vion A.C., Barbacena P., Fan J., Mathivet T., Fonseca C.G., Ragab A., Yamaguchi T.P. (2016). Non-canonical Wnt signalling modulates the endothelial shear stress flow sensor in vascular remodelling. Elife.

[23] Ola R., Künzel S.H., Zhang F., Genet G., Chakraborty R., Pibouin-Fragner L., Martin K., Sessa W., Dubrac A., Eichmann A. (2018). SMAD4 Prevents Flow Induced Arteriovenous Malformations by Inhibiting Casein Kinase 2. Circulation.

[24] Ola R., Dubrac A., Han J., Zhang F., Fang J.S., Larrivée B., Lee M., Urarte A.A., Kraehling J.R., Genet G. (2016). PI3 kinase inhibition improves vascular malformations in mouse models of hereditary haemorrhagic telangiectasia. Nat. Commun..

[25] Rochon E.R., Menon P.G., Roman B.L. (2016). Alk1 controls arterial endothelial cell migration in lumenized vessels. Development.

[26] Laux D.W., Young S., Donovan J.P., Mansfield C.J., Upton P.D., Roman B.L. (2013). Circulating Bmp10 acts through endothelial Alk1 to mediate flow-dependent arterial quiescence. Development.

[27] Alsina-Sanchís E., García-Ibáñez Y., Figueiredo A.M., Riera-Domingo C., Figueras A., Matias-Guiu X., Casanovas O., Botella L.M., Pujana M.A., Riera-Mestre A. (2018). ALK1 Loss Results in Vascular Hyperplasia in Mice and Humans Through PI3K Activation. Arter. Thromb. Vasc. Biol..

[28] Bry M., Kivelä R., Holopainen T., Anisimov A., Tammela T., Soronen J., Silvola J., Saraste A., Jeltsch M., Korpisalo P. (2010). Vascular endothelial growth factor-B acts as a coronary growth factor in transgenic rats without inducing angiogenesis, vascular leak, or inflammation. Circulation.

[29] Kivelä R., Bry M., Robciuc M.R., Räsänen M., Taavitsainen M., Silvola J.M., Saraste A., Hulmi J.J., Anisimov A., Mäyränpää M.I. (2014). VEGF-B-induced vascular growth leads to metabolic reprogramming and ischemia resistance in the heart. EMBO Mol. Med..

[30] Lähteenvuo J.E., Lähteenvuo M.T., Kivelä A., Rosenlew C., Falkevall A., Klar J., Heikura T., Rissanen T.T., Vähäkangas E., Korpisalo P. (2009). Vascular endothelial growth factor-B induces myocardium-specific angiogenesis and arteriogenesis via vascular endothelial growth factor receptor-1- and neuropilin receptor-1-dependent mechanisms. Circulation.

[31] Dewerchin M., Carmeliet P. (2012). PlGF: A multitasking cytokine with disease-restricted activity. Cold Spring Harb. Perspect. Med..

[32] Ylä-Herttuala S., Baker A.H. (2017). Cardiovascular Gene Therapy: Past, Present, and Future. Mol. Ther..

[33] Ozawa C.R., Banfi A., Glazer N.L., Thurston G., Springer M.L., Kraft P.E., McDonald D.M., Blau H.M. (2004). Microenvironmental VEGF concentration, not total dose, determines a threshold between normal and aberrant angiogenesis. J. Clin. Investig..

[34] Carmeliet P., Dor Y., Herbert J.M., Fukumura D., Brusselmans K., Dewerchin M., Neeman M., Bono F., Abramovitch R., Maxwell P. (1998). Role of HIF-1alpha in hypoxia-mediated apoptosis, cell proliferation and tumour angiogenesis. Nature.

[35] Grunewald M., Kumar S., Sharife H., Volinsky E., Gileles-Hillel A., Licht T., Permyakova A., Hinden L., Azar S., Friedmann Y. (2021). Counteracting age-related VEGF signaling insufficiency promotes healthy aging and extends life span. Science.

[36] Ziegler T., Horstkotte J., Schwab C., Pfetsch V., Weinmann K., Dietzel S., Rohwedder I., Hinkel R., Gross L., Lee S. (2013). Angiopoietin 2 mediates microvascular and hemodynamic alterations in sepsis. J. Clin. Investig..

[37] Arboleda-Velasquez J.F., Manent J., Lee J.H., Tikka S., Ospina C., Vanderburg C.R., Frosch M.P., Rodríguez-Falcón M., Villen J., Gygi S. (2011). Hypomorphic Notch 3 alleles link Notch signaling to ischemic cerebral small-vessel disease. Proc. Natl. Acad. Sci. USA.

[38] Jiang Q., Lagos-Quintana M., Liu D., Shi Y., Helker C., Herzog W., le Noble F. (2013). miR-30a regulates endothelial tip cell formation and arteriolar branching. Hypertension.

[39] Gaengel K., Niaudet C., Hagikura K., Siemsen B., Muhl L., Hofmann J.-A., Ebarasi L., Nystr+Âm S., Rymo S., Chen L.-A. (2012). The Sphingosine-1-Phosphate Receptor S1PR1 Restricts Sprouting Angiogenesis by Regulating the Interplay between VE-Cadherin and VEGFR2. Dev. Cell.

[40] Kupatt C., Hinkel R., Pfosser A., El-Aouni C., Wuchrer A., Fritz A., Globisch F., Thormann M., Horstkotte J., Lebherz C. (2010). Cotransfection of Vascular Endothelial Growth Factor-A and Platelet-Derived Growth Factor-B Via Recombinant Adeno-Associated Virus Resolves Chronic Ischemic Malperfusion: Role of Vessel Maturation. J. Am. Coll. Cardiol..

[41] Arsic N., Zentilin L., Zacchigna S., Santoro D., Stanta G., Salvi A., Sinagra G., Giacca M. (2003). Induction of functional neovascularization by combined VEGF and angiopoietin-1 gene transfer using AAV vectors. Mol. Ther..

[42] Red-Horse K., Das S. (2021). New Research Is Shining Light on How Collateral Arteries Form in the Heart: A Future Therapeutic Direction?. Curr. Cardiol. Rep..

[43] Lucitti J.L., Mackey J.K., Morrison J.C., Haigh J.J., Adams R.H., Faber J.E. (2012). Formation of the collateral circulation is regulated by vascular endothelial growth factor-A and a disintegrin and metalloprotease family members 10 and 17. Circ. Res..

[44] Rzechorzek W., Zhang H., Buckley B.K., Hua K., Pomp D., Faber J.E. (2017). Aerobic exercise prevents rarefaction of pial collaterals and increased stroke severity that occur with aging. J. Cereb. Blood Flow Metab..

[45] Zhang H., Prabhakar P., Sealock R., Faber J.E. (2010). Wide genetic variation in the native pial collateral circulation is a major determinant of variation in severity of stroke. J. Cereb. Blood Flow Metab..

[46] Lucitti J.L., Sealock R., Buckley B.K., Zhang H., Xiao L., Dudley A.C., Faber J.E. (2016). Variants of Rab GTPase-Effector Binding Protein-2 Cause Variation in the Collateral Circulation and Severity of Stroke. Stroke.

[47] Clayton J.A., Chalothorn D., Faber J.E. (2008). Vascular endothelial growth factor-A specifies formation of native collaterals and regulates collateral growth in ischemia. Circ. Res..

[48] Buschmann I., Pries A., Styp-Rekowska B., Hillmeister P., Loufrani L., Henrion D., Shi Y., Duelsner A., Hoefer I., Gatzke N. (2010). Pulsatile shear and Gja5 modulate arterial identity and remodeling events during flow-driven arteriogenesis. Development.

[49] Cristofaro B., Shi Y., Faria M., Suchting S., Leroyer A.S., Trindade A., Duarte A., Zovein A.C., Iruela-Arispe M.L., Nih L.R. (2013). Dll4-Notch signaling determines the formation of native arterial collateral networks and arterial function in mouse ischemia models. Development.

[50] Faber J.E., Storz J.F., Cheviron Z.A., Zhang H. (2021). High-altitude rodents have abundant collaterals that protect against tissue injury after cerebral, coronary and peripheral artery occlusion. J. Cereb. Blood Flow Metab..

[51] Schierling W., Troidl K., Troidl C., Schmitz-Rixen T., Schaper W., Eitenmüller I.K. (2009). The Role of Angiogenic Growth Factors in Arteriogenesis. J. Vasc. Res..

[52] Heil M., Ziegelhoeffer T., Wagner S., Fernandez B., Helisch A., Martin S., Tribulova S., Kuziel W.A., Bachmann G., Schaper W. (2004). Collateral artery growth (arteriogenesis) after experimental arterial occlusion is impaired in mice lacking CC-chemokine receptor-2. Circ. Res..

[53] van Royen N., Hoefer I., Bottinger M., Hua J., Grundmann S., Voskuil M., Bode C., Schaper W., Buschmann I., Piek J.J. (2003). Local Monocyte Chemoattractant Protein-1 Therapy Increases Collateral Artery Formation in Apolipoprotein E-Deficient Mice but Induces Systemic Monocytic CD11b Expression, Neointimal Formation, and Plaque Progression. Circ. Res..

[54] Troidl C., Jung G., Troidl K., Hoffmann J., Mollmann H., Nef H., Schaper W., Hamm C.W., Schmitz-Rixen T. (2013). The temporal and spatial distribution of macrophage subpopulations during arteriogenesis. Curr. Vasc. Pharmacol..

[55] Götze A.M., Schubert C., Jung G., Dörr O., Liebetrau C., Hamm C.W., Schmitz-Rixen T., Troidl C., Troidl K. (2020). IL10 Alters Peri-Collateral Macrophage Polarization and Hind-Limb Reperfusion in Mice after Femoral Artery Ligation. Int. J. Mol. Sci..

[56] Lauriol J., Keith K., Jaffr F., Couvillon A., Saci A., Goonasekera S.A., McCarthy J.R., Kessinger C.W., Wang J., Ke Q. (2014). RhoA signaling in cardiomyocytes protects against stress-induced heart failure but facilitates cardiac fibrosis. Sci. Signal..

[57] Dorn T., Kornherr J., Parrotta E.I., Zawada D., Ayetey H., Santamaria G., Iop L., Mastantuono E., Sinnecker D., Goedel A. (2018). Interplay of cell-cell contacts and RhoA/MRTF-A signaling regulates cardiomyocyte identity. EMBO J..

[58] Eitenmuller I., Volger O., Kluge A., Troidl K., Barancik M., Cai W.J., Heil M., Pipp F., Fischer S., Horrevoets A.J.G. (2006). The Range of Adaptation by Collateral Vessels After Femoral Artery Occlusion. Circ. Res..

[59] Limbourg A., Ploom M., Elligsen D., Sorensen I., Ziegelhoeffer T., Gossler A., Drexler H., Limbourg F.P. (2007). Notch Ligand Delta-Like 1 Is Essential for Postnatal Arteriogenesis. Circ. Res..

[60] Guan Y., Cai B., Wu X., Peng S., Gan L., Huang D., Liu G., Dong L., Xiao L., Liu J. (2017). microRNA-352 regulates collateral vessel growth induced by elevated fluid shear stress in the rat hind limb. Sci. Rep..

[61] Lei Z., van Mil A., Brandt M.M., Grundmann S., Hoefer I., Smits M., El Azzouzi H., Fukao T., Cheng C., Doevendans P.A. (2015). MicroRNA-132/212 family enhances arteriogenesis after hindlimb ischaemia through modulation of the Ras-MAPK pathway. J. Cell Mol. Med..

[62] Landskroner-Eiger S., Qiu C., Perrotta P., Siragusa M., Lee M.Y., Ulrich V., Luciano A.K., Zhuang Z.W., Corti F., Simons M. (2015). Endothelial miR-17∼92 cluster negatively regulates arteriogenesis via miRNA-19 repression of WNT signaling. Proc. Natl. Acad. Sci. USA.

